# Craniotomy for acute monitoring of pial vessels in the rodent brain

**DOI:** 10.1016/j.mex.2022.101694

**Published:** 2022-04-08

**Authors:** Refat Aboghazleh, Baraah Alkahmous, Evyatar Swissa, Saara Mansoor, Alon Friedman, Ofer Prager

**Affiliations:** a Department of Basic Medical Sciences, Faculty of Medicine, Al-Balqa Applied University, Al-Salt, Jordan; b Department of Internal Medicine, Prince Mohammed Bin Abdulaziz Hospital, Riyadh, Saudi Arabia; c Departments of Physiology and Cell Biology, Cognitive and Brain Sciences, Zlotowski Center for Neuroscience, Ben-Gurion University of the Negev, Beer-Sheva, Israel; d Department of Medical Neuroscience, Faculty of Medicine and Brain Repair Center, Dalhousie University, Halifax, Canada

**Keywords:** Blood-brain barrier, Craniotomy, Cerebrovascular imaging, Cranial window, Electrocorticography, BBB, blood-brain barrier, CNS, central nervous system, ECs, endothelial cells, ACSF, artificial cerebrospinal fluid, SpO2, blood oxygen saturation

## Abstract

A growing awareness for vascular contribution to pathogenesis of brain diseases increases the need for techniques that allow high-resolution imaging and quantification of changes in function and structure of cerebral microvessels. Cerebral vessels are very sensitive structures, making them vulnerable for injury. In addition, they are uniquely characterized with the blood-brain barrier, and an extra caution is required during procedures that involve engagement of cerebral vessels (i.e., craniotomy).

Using state of the art facilities, including 3D intravital microscope, we describe here in details:

• The steps and equipment required for drilling a craniotomy and removing of the dura, while keeping brain parenchyma and vessels intact. This enables long duration of live and direct monitoring of pial vessels and imaging of BBB permeability.
• We present the craniotomy procedure that relevant and compatible with imaging pial vessels and monitoring the blood-brain barrier in small rodents.

## Graphical abstract

**Figure:**
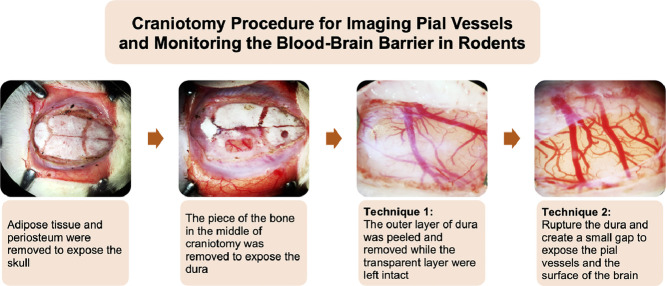

## Specifications table

**Table:** 

Subject Area:	Neuroscience
More specific subject area:	Imaging pial vessels and monitoring the blood-brain barrier
Method name:	Craniotomy
Name and reference of original method:	*N.A.*
Resource availability:	- https://www.rwdstco.com/ - https://www.finescience.com/en-US/ - https://www.zeiss.com/microscopy/int/products/stereo-zoom-microscopes/axio-zoom-v16.html

## Introduction

In many neurosurgical operations, such of brain tumors [4], drug resistant epilepsy [3], intracranial aneurysm and subarachnoid hemorrhage [5,8], a removal of a piece of the skull's bone (craniotomy) is required for accessing the brain directly. In animal research craniotomy is used during in vivo experiments [9,10,2], allowing extracellular and intracellular recording of neuronal activity, together with imaging of electrical signaling using fluorescent markers such as voltage sensitive dyes and calcium indicators. In recent years, with the better understanding of the role of other members of the neurovascular unit for normal brain function, including the vascular system and glia cells, new imaging techniques were developed to allow imaging of the cerebro-vasculature, and specifically the integrity of the blood-brain barrier (BBB). Parallel vascular imaging and electrophysiological recordings therefore enable studying in more detail neurovascular interactions in health and disease.

The BBB is a unique anatomical and physiological selective barrier in the central nervous system (CNS), separating the brain from the systemic circulation [11]. The BBB is composed of endothelial cells (ECs) that are inter-connected via tight and adherens junctions, thus limiting para-cellular passage and forcing molecules to pass through the endothelium using specialized routes [1]. The BBB controls and regulates the bi-directional passage of molecules between the blood and the brain, and maintains brain environment in homeostasis which essential for its normal function [1,11].

Since anatomically a rodent skull is in a very close proximity to the cortical surface (∼1 mm or less) and pial arterioles and capillaries are small in diameter (<100 mm), brain tissue is vulnerable for injuries, including disruption of the BBB and bleeding during craniotomy. Therefore, a critical step in these experimental procedures is a careful craniotomy and dura removal. Here we describe in detail the protocol we use.

## Procedure

All experiments were performed following institutionally approved protocols in accordance with the Canadian Council on Animal Care and were approved by the Dalhousie University Committee on Laboratory Animals.

### Instruments and equipment

- Scalpel handle (size 3) and surgical blade (gauge 10).
- Lidocaine (10%).
- Alcohol (90%) and distilled water.
- Artificial cerebrospinal fluid (ACSF), in mmol is: NaCl: 124, KCl: 3, CaCl₂: 2, MgSO₄: 2, NaHCO₃: 26, glucose: 10, pH 7.4 at 34-36°C, osmolarity 300 ± 10 mOsmol/l and carbonated with 95% O₂ and 5% CO₂ [6,9].
- Micro drill (RWD Life Science, SKU: 78001).
- Kimwipes (Kimtech Science, No. 34120).
- Micro tip cautery (Aaron Sterile High Micro Tip Cautery, SKU AA29).
- Curved serrated forceps (Fine Science Tools (F. S .T), No. 11051-10).
- Fine scissor (F. S. T, No. 14160-10).
- Micro scissor (F. S. T, No. 15000-03).
- Micro forceps (F. S. T, No. 11252-00 and 11274-20).
- Bone wax (Ethicon).
- Syringe (10 ml) filled with normal saline.
- Tissue clips.
- Needles.
- Cotton swabs.
- Synthomycine 5%.
- A surgical or intravital microscope (e.g. Axio Zoom, V16, Zeiss GmbH, or similar).
- Isoflurane, Isoflurane vaporizer and 99.9% oxygen tank.
- Heating pad (e.g. Physiotemp).
- Stereotaxic frame.
- Oximeter pod.

### The craniotomy procedure

**Fig. 1:**
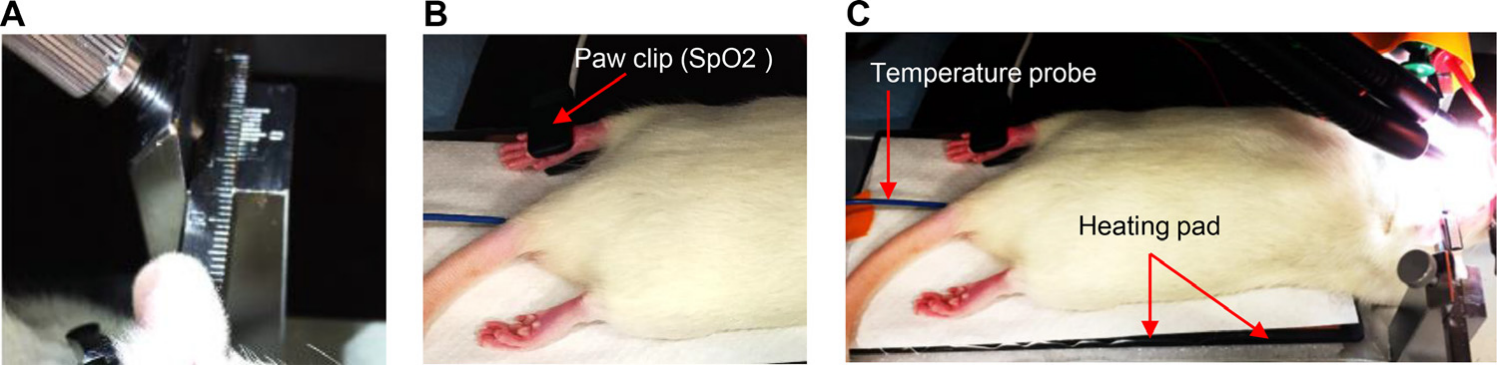
*Preparing a rat for a craniotomy*. (**A**) The animal mounted in a stereotaxic frame using ear bars. (**B**) Blood oxygen saturation (SpO2) monitored continuously using a paw clip connected to an animal oximeter pod. (**C**) The body temperature of animal is maintained at 37.3 °C using an electrical heating pad. Temperature probe inserted into the rectum of the animal.
(6) Blood oxygen saturation (SpO2) should be monitored continuously using a paw clip connected to an animal oximeter pod (ML325, ADInstruments, Sydney, Australia) and a PowerLab data acquisition device (PL3508, ADInstruments, Sydney, Australia) (Fig. 1B).
(7) It is important that core body temperature is maintained at 37.3 ± 0.2 °C using an electrical or water jacketed heating pad. For the electrical pad, insert the probe into the rectum of the animal and fix it to the tail with a tape. Monitoring the temperature is crucial as the animal is susceptible to hypothermia and could die (Fig. 1C).
(8) Adjust the light source.
(9) Apply synthomycine or any lubricant to the eyes of the animal.
(10) Around 10 min prior to the surgery, inject 0.3 ml of lidocaine 10% (dilute it up to 1 ml using normal saline) subcutaneously in the scalp. Distribute the injections over the area of incision.
(11) To clean the area of the incision, povidone-iodine scrub should be applied on the skin for one minute and then removed by swipes of 90% alcohol. This process should be repeated twice.
(12) Prior to the incision and every 15 min check the animal's pedal reflex. The withdrawal response to toe pinch must be absent. If there is any reflex, wait for additional 10 min and repeat it again. If the animal is showing positive reflex, then increase the isoflurane (e.g., from 1.5 to 2%), and then wait and check again.
(13) Using a scalpel handle (size 3) with surgical blade (preferred gauge 10), make a midsagittal incision (∼2.5 cm long) to expose the skull.
(14) Flip the edges of the incision to the right and left sides using tissue clips (Fig. 2A) or by using surgical thread to fix the tissue to the corners of the stereotaxic frame.

(1) Sedate the rat in isoflurane (3%) induction chamber until loss of toe-pinch withdrawal is observed (about 3–5 min) and animal has no pain, then shave the top of the rat's head using an electric shaver starting from the area between the eyes and moving back toward the neck.
(2) Clean the head of the rat using air canister and wipe it with distilled water to remove any remaining fur that may stick to the surface of the brain or impede dura removal.
(3) Check that the rat is sedated by pedal reflex (firm toe pinch), and if not, return it to the isoflurane induction chamber until the absence of withdrawal response to toe pinch.
(4) Prepare the surgery stage under the microscope in advance, and immediately transfer the rat and put it under deep anesthesia (1.5–2% of isoflurane for maintenance) and adjust the oxygen for 0.8–1 L/min. It is important to monitor the breathing pattern of the animal. If the rat is gasping and the chest and abdomen are moving heavily, then anesthesia is too deep and the concentration of isoflurane needs to be decreased. Alternatively, if the animal moves concentration of isoflurane should be increased. In addition, check that the nose cone is not creating pressure at the tip of the animal's nose.
(5) The animal should be mounted in a stereotaxic frame using ear bars, starting with the outside bar and then the bar closest to you. Ear bars should be inserted in the external auditory meatus (bone) and not against neck muscle so that the head will be fixed properly for drilling of the craniotomy without interrupting the breathing of the animal (Fig. 1A).

**Fig. 2:**
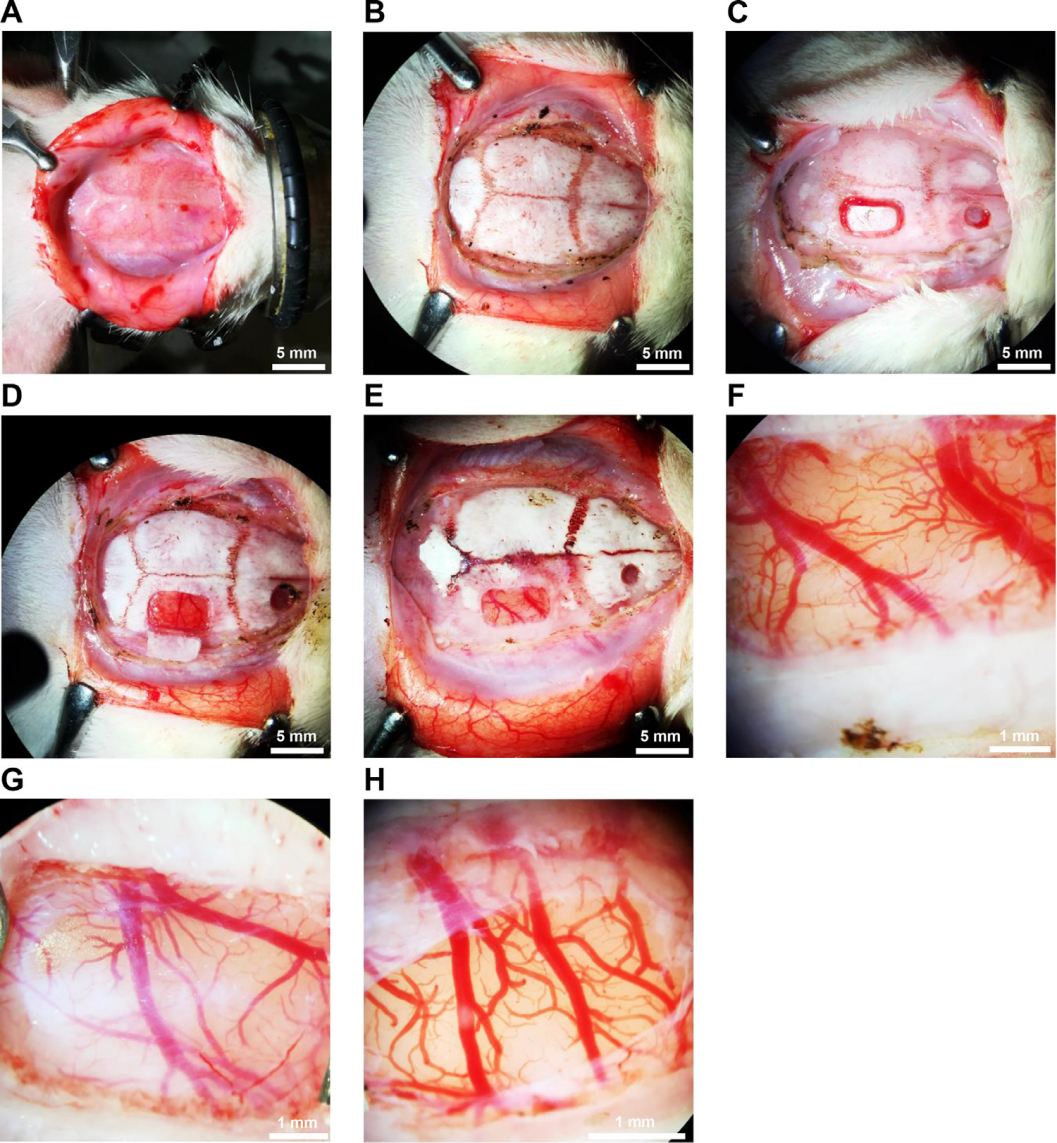
*Craniotomy for acute monitoring of pial vessels*. (**A**) A midsagittal incision made in the scalp. (**B**) Adipose tissue and periosteum were removed to expose the skull. The parietal window is drilled (**C**) and the piece of the bone in the middle of craniotomy is loose and can be removed with little effort (**D** and **E**). The outer layer of dura was peeled and removed while the transparent layer were left intact (**F** and **G**), in which homeostatic state of the brain is maintained. (**H**) Dura was ruptured, and small gap was created to expose the pial vessels and the surface of the brain.
(15) Remove adipose tissue using small scissors. Use a surgical blade to remove the periosteum by drawing a circle to expose the superior surface of the skull. Make sure to cut medial (central) to the temporalis muscle to avoid cutting its fibers. Remove any remaining tissue from the exposed bone and wash it using normal saline (Fig. 2B).
(16) To stop minor bleedings, use the cautery and the cotton swabs. Bone wax can also be used.
(17) The diameter or location for the cranial window depends on the goal of the experiment. The parietal bone provides the easiest access.
(18) Start drilling with a micro drill bit (0.7 mm).
  - It is highly recommended to drill under the guidance of a surgical or intravital microscope. Adjust the proper magnification of your microscope that enables you to see the whole area of your interest. You may need to adjust and change the magnification every so often during the surgery.
  - Drilling should be done under chilled ACSF (or saline) delivered often by a syringe to avoid heat injury.
  - Maintain the skull wet with cold ACSF (or saline) during the drilling.
  - Drill slowly, drilling too fast will increase the chance for a heat injury to the pial vessels and underlying cortex.
(19) While drilling, support your hand to avoid applying pressure on the drilling site and damaging the brain by penetrating the skull.
(20) Start etching the borders of the cranial window on the bone using the microdrill, then use this template to begin drilling. Do not drill on one side for extended periods of time, instead, move over the window template to avoid creating a heat injury or causing bleeding from the bone or dura.
(21) Continue drilling until the dura is reached or when the piece of the bone in the middle of craniotomy is loose (Fig. 2C) and can be removed with little effort using small, toothed forceps (Fig. 2D and E). Usually at this stage, minor bleeding can happen, and it can be stopped by rinsing the dura with ACSF and applying small cotton balls for a few minutes without creating any pressure.
(22) There are two techniques to expose the brain tissue:
  The first technique is to keep the dura intact and use a tip of a small needle (of insulin syringe or the needle from a cannula 24-gauge) to make it thinner. This technique preserves the intracranial pressure of the brain and maintains the homeostatic state of the brain with its own cerebrospinal fluid. It is important to create a microhook on the tip of the needle (use forceps to bend) to scratch the outer layer of the dura. Create a small longitudinal shallow groove in the dura by scratching the first layer of the dura slowly and gently moving from medial (central) to lateral. The dura is composed of two layers, an outer layer rich in vessels and an inner transparent layer. In this procedure, the outer layer can be identified through the shallow groove and removed. Using fine forceps grasp the edges of the outer layer of the dura from the groove and peel them slowly to the right and left along the groove (Fig. 2F and G) until you obtain the amount of exposure required.
  The second technique is to rupture the dura using the needle. Try to choose a location where there are no pial vessels located beneath it. Open a small gap in the dura, then using small forceps or a micro incisor cut the dura (Fig. 2H). The exposed brain should be perfused and continuously nourished by artificial cerebrospinal fluid at rate of 1.0 ml/min [7].
  Compared to prior studies where the open-window technique, i.e., craniotomy, was presented only in general, here we describe the procedure in detail, and cautiously explain its steps. This is essential and can allow to learn how to perform the technique without damaging the brain and enable doing direct and sensitive in vivo imaging of pial microvessels.

## Declaration of Competing Interest

The authors declare that they have no known competing financial interests or personal relationships that could have appeared to influence the work reported in this paper.
